# A Text Message Delivered Smoking Cessation Intervention: The Initial Trial of TXT-2-Quit: Randomized Controlled Trial

**DOI:** 10.2196/mhealth.2522

**Published:** 2013-07-30

**Authors:** Beth Bock, Kristin Heron, Ernestine Jennings, Kathleen Morrow, Victoria Cobb, Joshua Magee, Joseph Fava, Christopher Deutsch, Robert Foster

**Affiliations:** ^1^Alpert Medical School, Brown UniversityCenters for Behavioral and Preventive MedicineProvidence, RIUnited States; ^2^Survey Research CenterPennsylvania State UniversityUniversity Park, PAUnited States; ^3^The Miriam HospitalCenters for Behavioral and Preventive MedicineProvidence, RIUnited States; ^4^University of CincinnatiDept. of Family and Community MedicineUniversity of CincinnatiCincinnati, OHUnited States; ^5^The Miriam HospitalCenters for Behavioral and Preventive MedicineThe Miriam HospitalProvidence, RIUnited States; ^6^Live Inspired LLCBethesda, MDUnited States

**Keywords:** smoking cessation, tobacco, texting, text messaging, mobile health, mHealth, health communications

## Abstract

**Background:**

Mobile technology offers the potential to deliver health-related interventions to individuals who would not otherwise present for in-person treatment. Text messaging (short message service, SMS), being the most ubiquitous form of mobile communication, is a promising method for reaching the most individuals.

**Objective:**

The goal of the present study was to evaluate the feasibility and preliminary efficacy of a smoking cessation intervention program delivered through text messaging.

**Methods:**

Adult participants (N=60, age range 18-52 years) took part in a single individual smoking cessation counseling session, and were then randomly assigned to receive either daily non-smoking related text messages (control condition) or the TXT-2-Quit (TXT) intervention. TXT consisted of automated smoking cessation messages tailored to individual’s stage of smoking cessation, specialized messages provided on-demand based on user requests for additional support, and a peer-to-peer social support network. Generalized estimating equation analysis was used to assess the primary outcome (7-day point-prevalence abstinence) using a 2 (treatment groups)×3 (time points) repeated measures design across three time points: 8 weeks, 3 months, and 6 months.

**Results:**

Smoking cessation results showed an overall significant group difference in 7-day point prevalence abstinence across all follow-up time points. Individuals given the TXT intervention, with higher odds of 7-day point prevalence abstinence for the TXT group compared to the Mojo group (OR=4.52, 95% CI=1.24, 16.53). However, individual comparisons at each time point did not show significant between-group differences, likely due to reduced statistical power. Intervention feasibility was greatly improved by switching from traditional face-to-face recruitment methods (4.7% yield) to an online/remote strategy (41.7% yield).

**Conclusions:**

Although this study was designed to develop and provide initial testing of the TXT-2-Quit system, these initial findings provide promising evidence that a text-based intervention can be successfully implemented with a diverse group of adult smokers.

**Trial Registration:**

ClinicalTrials.gov: NCT01166464; http://clinicaltrials.gov/ct2/show/NCT01166464 (Archived by WebCite at http://www.webcitation.org/6IOE8XdE0).

## Introduction

It is estimated that 19.3% (45.3 million) of US adults are smokers, with higher rates among younger adults and individuals with lower socioeconomic status [1]. Smoking or exposure to second-hand smoke kills more than 440,000 people in the USA every year, and is associated with numerous chronic health conditions, which results in nearly $100 billion in healthcare costs and productivity losses annually [2,3]. Estimates suggest that 69% of smokers want to quit, and in 2010, more than half of all smokers attempted to quit [1]. Although evidence-based behavioral smoking treatments exist, research suggests that these resources are used by less than 10% of smokers attempting to quit [4,5]. Frequently cited barriers to utilizing behavioral interventions include the cost, time commitments, and logistics (eg, travel, scheduling appointments) associated with these treatments [6]. To effect significant reductions in smoking rates, innovative interventions and delivery systems are needed that reach smokers effectively and efficiently [7].

One emerging method that may help overcome these barriers to treatment is the use of mobile communication technologies, or mHealth treatment programs. The use of mobile technology, such as mobile phones, smartphones, and tablet devices to deliver health-related interventions is a rapidly expanding area of research and practice [8-11]. With more than 80% of adults in the USA-across various demographic groups-owning mobile phones [12], the majority of smokers can be reached for smoking cessation treatment using mobile technology. Previous research suggests (“landline”) telephone counseling for smoking cessation is efficacious [13], is acceptable to smokers, and in many cases is the preferred mode of treatment as compared with face-to-face behavioral counseling [13,14].

As with landline telephones, mobile phones can be used to provide behavioral counseling, but offer the additional benefit of allowing for SMS text messaging (short message service, SMS) as well. SMS text messaging-based mHealth interventions that use texting are becoming popular because of the ease-of-use and low cost of SMS text messaging. This intervention modality also offers several benefits over face-to-face or telephone-based behavioral interventions, primarily because mobile technology allows interventions to be delivered to people in everyday settings and in real-time. This allows for the content and timing of messages to be individually tailored to individuals. For example, text messages encouraging continued abstinence from smoking can be provided at times when patients report they are in need of additional support (eg, when having cravings, at times when they typically smoke). The use of mobile technology can allow for multiple daily contacts over longer periods of time (ie, several text messages per day). This increased intensity and tailoring of interventions may improve adherence to self-help materials, resulting in higher quit rates [15-18].

To date, there have been several interventions designed using SMS text messaging to deliver smoking cessation interventions. Although many studies have evaluated short-term treatment outcome for smokers either using single group [19,20] or randomized designs [21-23], there are fewer SMS text messaging-based smoking cessation programs that have carefully examined long-term outcome data (≥6 months) in randomized trials [24,25]. In the past several years, two Cochrane reviews have been conducted evaluating the short-term [26] and longer-term [27] efficacy of SMS text messaging-based smoking cessation interventions. The authors have concluded that although there is heterogeneity among study results, overall there is a benefit of mobile phone-based smoking cessation interventions.

The goal of the present work was to develop and evaluate the feasibility and preliminary efficacy of a smoking cessation intervention program delivered through SMS text messaging. The content and structure of this program was designed based on evidence-based intervention guidelines for smoking treatment programs, while also incorporating feedback from potential end users regarding their preferences. Formative work was conducted to assess user preferences for the intervention features, content and delivery schedules, and is reported elsewhere [28]. In the present study, we report on the intervention feasibility and acceptability of this intervention method, and present data from an initial study that included follow-up assessments thorough six months post-treatment.

## Methods

### Participants

Adult smokers were recruited to participate in this study. To be eligible for this study, individuals had to meet the following inclusion criteria: (1) current daily smoker, (2) interested in quitting smoking in the next 30 days, (3) have a mobile phone with SMS text messaging capability, and (4) use SMS text messaging at least once monthly.

### Measures

#### Baseline Measures

Smoking history variables were assessed, including age when started smoking, current number of cigarettes smoked per day, number of quit attempts in the last year, and methods used to attempt to quit. Nicotine dependence was measured using the 6-item Fagerstrom Test for Nicotine Dependence (FTND) [29]. Scores can range from 0-10 with higher numbers indicating greater levels of nicotine dependence. Symptoms of nicotine withdrawal were measured using the Mood and Physical Symptoms Scale (MPSS) [30]. The MPSS uses a 5-point rating scale assessing mood, irritability, restlessness, difficulty concentrating, and hunger, and has an additional item using a 6-point scale to assess urges to smoke. We also used a 16-item instrument derived from work by Hughes and Hatsukami [31] that assessed the degree to which participants had experienced typical symptoms of nicotine withdrawal during previous quit attempts. Readiness and confidence in ability to quit smoking were assessed using two items. Participants used a 10-point scale to rate readiness to quit (1=not ready at all, 10=extremely ready) and confidence (1=very low confidence, 10=extremely confident). Readiness to quit smoking was also assessed using the stages of change measure, with participants indicating their number of quit attempts in the last year and how seriously they are considering quitting (within next 30 days, within next 6 months, not thinking of quitting). This measure was used to identify a person’s current stage of change for quitting smoking [32]. The Center for Epidemiologic Studies Depression Scale (CESD-10) [33] was used to measure depressive symptoms. Participants rate the extent to which they experience specific feelings or engaged in behaviors during the previous week with responses ranging from rarely (0) to most or all of the time, 5-7 days (3). Scores range from 0 to 30 with higher scores indicating greater depressive symptoms.

#### Outcome Measures

Outcome measures were administered mid-intervention (week 4), post-intervention (week 8), and at 3 and 6 months follow-up assessments. During these times, participants reported their smoking status, readiness, and confidence in quitting (or remaining quit), and symptoms related to nicotine withdrawal. Smoking status included 7-day point prevalence abstinence, the primary outcome variable, and 24-hour point prevalence abstinence. Readiness and confidence in quitting and nicotine withdrawal symptoms were assessed using the measures described above. Feasibility and acceptability of the program were assessed by recruitment numbers, participant retention through the treatment program and final follow-up assessment, and results of a program satisfaction survey.

### Intervention Design and Components

Formative work which included several focus groups with young adult smokers was conducted to inform the development of the intervention design and content [28]. The intervention, including both the SMS text messaging and individual counseling session, was modeled after national treatment guidelines [34], and guided by Social Cognitive Theory [35,36] and the stages of change model [37]. All intervention content (including the text message and counseling session content) was developed and reviewed by PhD-level clinical psychologists with expertise in smoking cessation treatment.

### Individual Counseling Session

At study enrollment, all participants also took part in an individual 30-minute counseling session, led by a PhD-level clinical psychologist. Participants chose the format of the session (ie, in-person, telephone, Google chat, or Skype), and were provided a copy of a quit smoking guide published by the American Lung Association [38]. During the session, the counselor led a discussion based upon key sections of the guide, including reasons for quitting, perceived importance of quitting, and confidence in one’s ability to quit, identifying obstacles to quitting, preparing for quit day, and planning strategies to aid quitting. Participants were encouraged to continue using the guide during their participation in the intervention.

### Text Message Intervention Content and Design

The intervention was designed to accommodate participants who were at various stages of quitting and to allow for different trajectories to abstinence. Four tracks were created that included “Not Ready”, “Prepare”, “Quit”, and “Relapse”. The “Not Ready” track was designed for individuals who wished to quit smoking in the next 30 days, but who were not ready to set a quit date. Messages in the “Not Ready” track consisted of once daily messages delivered for up to 14 days and were aimed at enhancing motivation to quit. The “Prepare” track was designed for individuals who set a targeted quit day within the next 14 days, and consisted of twice daily messages that included tips and advice on obtaining support for quitting, medications to aid quitting, dealing with nicotine addiction, coping with stress, problem solving and self-monitoring, and motivational messages. More than 200 messages were generated to address the following categories: social support, problem solving, decision making, motivational support, behavioral tips, information about smoking cessation medications, and addiction education. Participants received messages that spanned these categories to provide broad coverage of topics, and to ensure messages were not duplicated.

To avoid redundancy, variations on these messages were created for a “Prepare-2” track, which was delivered to those who failed to quit on quit day or relapsed. The “Quit” track contained messages delivered 4 times daily for 2 weeks, then twice daily for 4 weeks (6 weeks in total). These messages addressed the same general topics noted above, but were tailored to be appropriate for individuals who were currently engaged in quitting smoking.

At study enrollment, participants randomized to the intervention (TXT) were assigned either to the “Prepare” or “Not Ready” tracks depending on whether they set a target quit date. Those who remained in the “Not Ready” track for all 14 days without setting a quit date were called by the study counselor and encouraged to set a quit date. At an individual’s designated quit day, participants were moved into the “Quit” track. During the first week following the quit day, participants answered texted questions regarding whether they had been able to quit. Those who did not quit on their quit date or who relapsed were asked whether they wished to set a new date. Those setting a new quit date within 14 days were moved into the “Prepare” track, the remaining participants were moved into the “Not Ready” track. Participants could text the key words “Prepare”, “Quit”, “Not Ready”, and “Relapse” at any time during the program to move themselves into the appropriate track for their experience with quitting. For example, an individual in “Prepare” track who decided to quit several days before his/her designated quit day could text “Quit” to move himself/herself immediately into that part of the program. A participant in the “Quit” part of the program could text “Relapse” if he/she was smoking again and could then choose to begin the “Not Ready” or “Prepare” track.

### Additional Intervention Features

In our formative work, focus group participants strongly supported being able to receive messages “on demand” at times when they were experiencing a craving. This feature was included in the intervention; participants could text “Crave” and would receive an SMS text message back with a tip for coping with cravings. In addition, after quitting, participants reporting that they had “slipped” and smoked a cigarette, would receive SMS text messages immediately and twice daily for 3 days targeting coping and getting back on track with quitting. After a “slip” they were texted to report on their abstinence status and received tailored messages depending on their response.

### Control Condition

The SMS text messaging intervention was compared to a control condition. Participants who were randomly assigned to the control group (Mojo) received the same initial counseling session followed by 8 weeks of daily non-smoking-related motivational texts (eg, “It takes just one positive step to begin the journey out of a difficult rut. Step out today!”).

### Procedures

This study was conducted in the research facilities of the Miriam Hospital, which is affiliated with the Alpert School of Medicine at Brown University, and was approved by the Institutional Review Board prior to initiating recruitment. Participants were recruited between January and June 2011 through advertisements in local media outlets (Internet sites, radio programs). Interested individuals called or texted our study phone number for more information. The study research assistant (RA) reached callers by voice phone, provided a brief description of the study (pre-screening introduction), and screened potential participants for eligibility (see Participants section above for eligibility criteria). Eligible individuals were then scheduled for an in-person orientation visit during which they were given more information about the program, provided written informed consent, and took part in a single in-person smoking cessation counseling session. Simple randomization was used to assign participants to each group via a computerized random number generator. Random assignments were placed in a sealed envelope by the study RA prior to each counseling appointment. The RA delivered the randomization assignment to the study participant immediately after completion of the counseling session.

Over a period of 3 months, a total of 7 participants were enrolled and randomized using these procedures. Slow recruitment and high attrition rates prior to attending orientation prompted a change in recruitment methods.

We developed a Web portal that provided the pre-screening introduction to the study. Interested individuals clicked through to a second page that presented an online screener to determine the individual’s eligibility. Eligible individuals were then presented with an online consent form to sign electronically. The online consent included a brief quiz to ensure that individuals understood the primary points of the trial. After signing consent, the participant provided identifying and contact information, and completed an online baseline assessment. At the conclusion of the assessment, the program used simple randomization to assign individuals to the study arms and then presented an online Google calendar to schedule their counseling session. Study staff ran a series of 10 mock participants with differing answers to questions in order to test the accuracy of data collected on the Web portal before launching. New advertisements were developed to include the option of accessing the study website directly, in addition to calling or texting our staff. After implementation, 51 participants were recruited and randomized over 21 days using these website-based procedures.

**Figure 1:**
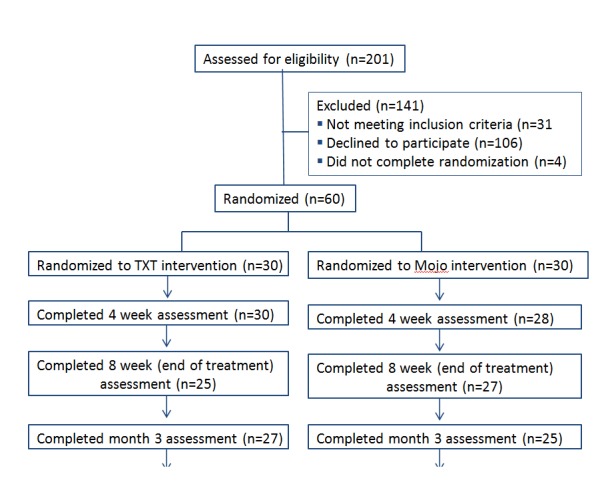
Recruitment Flow Diagram.

Once enrolled, participants completed the individual counseling session and began receiving either the intervention or control SMS text messages on their personal mobile phones for the following 8 weeks. At the mid-point (week 4) and end (week 8) of the intervention, all participants completed the outcome measures described above using the website. The study RA sent email reminders to participants when assessments were due reminding them to complete the online questionnaires and provided a link to the online questionnaires. The study RA conducting assessments and the counselors were blind to participant randomization assignment. Participants completed the outcome measures 3 and 6 months after completing the intervention (see Figure 1, Consort Diagram).

### Statistical Analyses

Frequency distributions, means, and standard deviations were used to characterize the overall sample. Chi-square tests and analysis of variance was used to compare groups for comparability on baseline demographic and smoking history variables. The primary smoking outcome analysis examined the 7-day point prevalence abstinence from smoking using the generalized estimating equations (GEE) approach of Zeger and Liang [39] with robust standard errors, using Proc GENMOD within SAS 9.3 for Windows. The model for this outcome analysis was a 2 (treatment groups)×3 (time points) repeated measures design that was fit using an autoregressive working correlation structure on data analyzed from an intention-to-treat (ITT) perspective in which missing participants were counted as smoking. Smoking outcomes were analyzed using odds ratios comparing TXT and Mojo (control) groups. A secondary smoking outcome analysis also used the GEE methodology to examine 24-hour point prevalence abstinence. Further secondary analyses used analysis of variance to evaluate for change in several potential mediating variables at 8 weeks, 3 months, and 6 months follow-up, among respondents who provided complete data at those assessment points.

## Results

### Participant Characteristics

Randomized participants (N=60) averaged 30.7 years of age (SD 9.0; range 18-52), and 43% (25/60) of them were male participants. Participants were primarily non-Hispanic white (39/60, 66%) or Black (11/60, 19%), with 7% (4/60) bi/multi-racial or unsure, 2% (1/60) Hispanic White, and 7% (4/60) did not respond. With respect to ethnicity, 19% (11/60) were Hispanic/Latino. Most had completed some college education (21/60, 36%) or had graduated from college (15/60, 26%), with 21% (12/60) graduating from high school, while 9% (5/60) had not graduated from high school, and 9% (5/60) did not respond. Most participants worked part time (21/60, 35%) (≤35 hours/week) or full time (18/60, 31%), while 26% (15/60) were unemployed and 9% (5/60) did not respond. Total household income was less than $25,000 for half (50%) of the participants.

On average, participants were 16 years old (SD 2.9) when they first started regular (daily) cigarette smoking. At the start of the study, participants smoked on an average of 16.3 cigarettes/day (SD 8.3; range 4-40). Participants had made an average of 4.1 (SD 3.8) serious quit attempts in their lives. Of the 53 participants who responded to a temporal intention to quit item, 94% noted at baseline that they were planning to quit in the next 30 days. Baseline FTND scores averaged 4.9 (SD 2.5), suggesting moderate nicotine dependence. Baseline CESD scores averaged 10.4 (SD 5.9), and over one third of participants (35%) had CESD scores above 11, indicative of significant levels of depressive symptoms [33]. There were no significant differences between treatment groups on any demographic or baseline smoking history variables.

### Smoking Outcome

Table 1 presents the percent of participant who quit smoking by group at each assessment time point.

A 2 (treatment groups)×3 (time points) GEE repeated measures analysis examined the effects of the TXT intervention versus Mojo for differences in 7-day point prevalence abstinence. There was a significant main effect for treatment group (*P*=.02) with higher odds of 7-day point prevalence abstinence for the TXT group compared with the Mojo group (OR 4.52, 95% CI 1.24, 16.53). There were no significant effects for time (*P*=.34) or the time × treatment group interaction (*P*=.60). While the overall main effect was significant, contrast estimates did not find specific differences between TXT versus Mojo at week 8 (23.3% vs 10.7%, OR 2.54, 95% CI 0.59, 10.99), month 3 (16.7% vs 3.6%, OR 5.40, 95% CI 0.59, 49.47), or month 6 (20.0% vs 3.6%, OR 6.75, 95% CI 0.76, 60.15), likely due to reduced statistical power at any individual time point. We also evaluated for treatment group differences in 24-hour point prevalence in a similar manner, using a 2 (treatment groups)×3 (time points) GEE repeated measures analysis, but found no significant effects for group (*P*=.11), time (*P*=.23), or the time × treatment group interaction (*P*=.88).

### Secondary Smoking-Related Outcome

Although this was an initial (ie, pilot) study and was not powered to detect mediation effects, we conducted hypothesis-generating exploratory analyses of additional smoking-related variables. Analysis of variance was used to examine for change in participants’ confidence to remain quit, readiness for change, level of depression, and nicotine withdrawal symptoms at the 8-week, 3-month and 6-month follow-ups. From baseline to the end of treatment (8 weeks), individuals in the TXT group tended to show increase in confidence in remaining quit (M 0.19, 3% increase) compared with those in the Mojo group (M -0.89, 12% decrease), although this difference was not significant. At 3 months follow-up, there was a trend (*P*=.07) for the MPSS Urge scale to be reduced among TXT participants compared with Mojo participants. During the 6 months follow-up, there was a significant improvement in MPSS mood symptoms of nicotine withdrawal (*P*=.03) among the TXT participants as compared with Mojo participants. No significant changes were seen in readiness to change or depression (CESD) scores.

**Table 1:** Smoking status by group for 7-Day and 24-Hour ITT at 8 weeks, 3 months, and 6 months.

				Total n (%)	Txt2Quit n (%)	Mojo n (%)
Sample size				60	30	30
**7-Day quit: 8 Weeks**						
	Yes			10 (17.2%)	7 (23.3%)	3 (10.7%)
	No			48 (82.8%)	23 (76.7%)	25 (89.3%)
**7-Day quit: 3 Months**						
		Yes		6 (10.3%)	5 (16.7%)	1 (3.6%)
		No		52 (89.7%)	25 (83.3%)	27 (96.4%)
**7-Day quit: 6 Months**						
			Yes	7 (12.1%)	6 (20.0%)	1 (3.6%)
			No	51 (87.9%)	24 (80.0%)	27 (96.4%)
**24-Hour quit: 8 Weeks**						
		Yes		12 (20.7%)	8 (26.6%)	4 (14.3%)
		No		46 (79.3%)	22 (73.3%)	24 (85.7%)
**24-Hour quit: 3 Months**						
			Yes	7 (12.1%)	5 (16.7%)	2 (7.1%)
			No	51 (87.9%)	25 (83.3%)	26 (92.7%)
**24-Hour quit: 6 Months**						
			Yes	8 (13.8%)	6 (20.0%)	2 (7.1%)
			No	50 (86.2%)	24 (80.0%)	26 (92.7%)

### Intervention Feasibility and Acceptability

One of the goals of this trial was to establish the feasibility and acceptability of a text message-based smoking cessation intervention. During the first 3 months of recruitment for this study, we used traditional recruitment and intervention delivery strategies (telephone screening, in-person orientation, face-to-face counseling), but found that with these methods less than 8% of individuals who were screened were eligible and enrolled, and we were only able to randomize 7 participants during this 3 months period (see Table 2). This very low recruitment yield prompted us to switch to online recruitment methods as described previously. Although approximately half (50.8%) of individuals who accessed the website abandoned it before providing consent, 92% of the remaining individuals completed study enrollment and were randomized, resulting in a 41.7% yield from all initial contacts and screens, as shown in Table 2.

Another unique aspect of this intervention was that we provided participants with a choice regarding the mode of delivery of the initial counseling session. We found that counseling visits were accomplished by voice-phone (56/100, 56%), in-person (18/100, 18%), Google chat (20/100, 20%), or Skype (6/100, 6%). Over half (61/100, 61%) of all participants used the online Google calendar to schedule their counseling session. End of treatment (week 8) satisfaction ratings showed that nearly all participants said they were *satisfied* (41/100, 41%) or *very satisfied* (48/100, 48%) with the program, and 63% (63/100) of participants said that the program met their expectations. Most participants said that the program was helpful (40/100, 40%) or very helpful (45/100, 45%). However, 60% (60/100) of participants did not use the group messaging (“help”) feature. Of those who did, 72% (72/100) said it was *easy* or *very easy* to use. Participants’ feedback indicated strong appreciation of the social networking feature. As an illustrative example, one participant reported: “Yes, I think that it helps me a lot…people to talk with when you would have normally smoked a cig.” Nearly one third of participants (n=17) used the peer-to-peer network for social support during their quit attempt. The number of messages sent by any one participant ranged from 2 to 177 (M 39.7, SD 54.0).

**Table 2:** Recruitment yield using traditional voice phone, SMS text messaging, and online methods (N=60).

	Standard recruitment 3 months		Online recruitment 21 days
	Voice phone, n	Txt Msg, n	Online, n
Total inquiries	96	51	155
Unable to contact	36	28	0
No longer interested	9	0	28
Screened	51	23	127
Ineligible	11	9	11
Eligible	40	14	116
Not interested, in-person orientation	16	11	-
Attended orientation	24	3	-
No-show for orientation	18	0	-
Lost before consent	2	0	59
Enrolled/signed consent	4	3	57
Randomized	4	3	53
Yield from initial contacts (%)	4.1	5.8	34.1
Yield from total screened (%)	7.8	13.0	41.7
Percentage of total sample (%)	6.7	5.0	88.3

## Discussion

### Principal Findings

Although this study was designed to develop and provide initial testing of the TXT-2-Quit system, significant differences were found between treatment groups, with individuals randomized to the intervention group showing higher point-prevalence abstinence rates vs the comparison group. These results are especially encouraging given that all control participants received an individual smoking cessation counseling session and at least one daily SMS text message for the duration of the 8-week intervention. Although participants in the TXT condition received more “contact” with researchers via multiple daily SMS text messages (albeit through an automated system, not direct personal contact), this well-matched control condition provided a more rigorous test than usual care. As such, finding significant treatment effects indicates that the effects of this intervention are potentially quite robust. Although preliminary, there was also an indication that participants receiving the text message intervention reported lower levels of nicotine withdrawal symptoms than those in the control condition. Future work that is statistically powered for secondary outcome variables should more vigorously evaluate factors that could serve as mediators or moderators of treatment efficacy, including self-efficacy for quitting, nicotine withdrawal symptoms, readiness for change, and depression.

One of the strengths of this study is that a relatively diverse sample was recruited to participate and found the intervention appealing, including a substantial number of ethnic and racial minorities and participants of many education levels. While over half of participants had at least some college (13-16 years formal education) education, over one-quarter had 12 years or less formal education. These statistics speak to the potential of mobile phones and SMS text messaging in the United States and many other countries for overcoming barriers to treatment access and usage.

While we initially sought to develop and test this intervention with younger adults (age <35 years), we eliminated the upper age limit after receiving many enquiries from individuals in their 40s and 50s. According to the Pew Internet and American Life Project, SMS text messaging is increasingly common among middle age and older adults, with 72% (72/100) of mobile phone owners aged 50-64 and 34% (34/100) of those aged 65+ years reporting using their mobile phone to send or receive SMS text messages [40]. We were unable to examine the extent to which age may act as a moderator of intervention efficacy because of the relatively small sample size for this initial study. However, it is possible that as SMS text messaging is becoming a more acceptable mode of communication among middle age and older adults that age may have a smaller moderating effect that in the past, although this remains an important area for future research.

Recruitment for this intervention was far more successful when the entire recruitment and treatment process was mobile phone and Web-based. Use of our traditional method of recruitment including in-person visits for orientation and consent together with an initial face-to-face smoking cessation counseling visit resulted in very slow recruitment and high rates of loss of eligible individuals. Both recruitment yield and speed were greatly improved when all processes were revised to be congruent with the way that the target audience uses technology.

This intervention was developed based on feedback from focus groups consisting of likely end users (ie, smokers who use SMS text messaging) [28]. A peer-to-peer network, which allowed participants to communicate and encourage each other, was created at the behest of focus group participants. However, we found that this feature was used by less than half of study participants. As designed, the social support feature was only accessed by individuals who requested “help” from others. It may be that some individuals do not need additional social support through this type of intervention or that they already receive (or think they receive) sufficient support from other social networks. Some people may also be hesitant to admit they need help, or tend to underestimate the degree to which seeking help from peers would improve their chances of quitting. This may need to be tested by making the social support network a more integrated (non-optional) part of the intervention in order to better evaluate the utility of including peer networks in SMS text message-based smoking cessation programs.

### Limitation

This study built upon previous interventions using SMS text messaging by using less study staff support, greater reliance on participants for providing group support, having a rigorous control condition which received daily SMS text messaging (non-smoking related) and the same individual smoking cessation counseling session as the intervention group. At the same time, there were also limitations that can be addressed by future research. First, results from pilot studies are inherently less reliable due to the small number of participants [41]; therefore, these findings will need replication in a larger trial to verify reliability of the results. Second, given that this was a pilot study, we did not biochemically verify abstinence from smoking, but instead relied on self-report. When this and other interventions are tested in larger clinical trials, objective measures of outcome should be used in order to reduce biases associated with self-reported abstinence from smoking. Third, larger scale studies are needed to provide sufficient statistical power to examine factors that may act as mechanisms of action as well as factors that may moderate intervention efficacy. Fourth, more thorough integration of peer-to-peer social networking within an SMS text message intervention could further enhance engagement with and benefits resulting from this type of treatment.

### Conclusions

Despite limitations, these findings add to the small but growing body of literature on text messaging interventions. Several studies have examined using SMS text messaging for smoking cessation, either as a stand-alone intervention [24,25] or as part of a multi-component intervention [21,42], or as part of a relapse prevention intervention [23]. In general, these studies have shown efficacy for smoking cessation interventions delivered by SMS text messaging. However, existing studies have been quite diverse in the intensity and length of the intervention as well as use of additional features (eg, face-to-face counseling and use of website or phone counseling to supplement the SMS text messaging intervention). As with computer and Web-based programs [43], it seems likely that programs using text messaging, either exclusively or as part of a larger intervention, will provide some level of efficacy for some smokers. Much work is needed to determine the effects that can be achieved with text alone versus text as a support to a larger intervention, as well as for which populations and under what circumstances these programs will be efficacious.

## Multimedia Appendix 1

CONSORT-EHEALTH checklist V1.6.2 [44].

## Abbreviations

CESD: Center for Epidemiologic Studies Depression Scale
FTND: Fagerstrom Test for Nicotine Dependence
GEE: generalized estimating equations
ITT: intention-to-treat
MPSS: Mood and Physical Symptoms Scale
RA: research assistant
