# Supplementary material for: A Text Message Delivered Smoking Cessation Intervention: The Initial Trial of TXT-2-Quit: Randomized Controlled Trial
Source: JMIR Mhealth Uhealth. 2013 Jul 30;1(2):e17. doi: 10.2196/mhealth.2522 (PMC4114437; doi:10.2196/mhealth.2522)
Supplement: Supplementary file 1 [file mhealth_v1i2e17_app1.pdf]

CONSORT-EHEALTH Checklist V1.6.2 Report

(based on CONSORT-EHEALTH V1.6), available at [http://tinyurl.com/consort-ehealth-v1-6].

2522

Date completed

6/27/2013 10:09:47

by

Beth Bock

A Text Message Delivered Smoking Cessation Intervention: The Initial trial of TXT-2-Quit, a Randomized, Controlled Trial

TITLE

1a-i) Identify the mode of delivery in the title

The title includes that it is text message-based:
Title: A Text Message Delivered Smoking Cessation Intervention: The Initial trial of TXT-2-Quit, a Randomized, Controlled Trial

1a-ii) Non-web-based components or important co-interventions in title

All participants received an initial counseling session by a trained counselor prior to the intervention and control, which were entirely delivered via text message without other components. "Counseling visits were accomplished by voice-phone (56%), in person (18%), Google Chat (20%), or Skype (6%)."

1a-iii) Primary condition or target group in the title

The title includes that it is focused on smoking cessation patients:
Title: A Text Message Delivered Smoking Cessation Intervention: The Initial trial of TXT-2-Quit, a Randomized, Controlled Trial

ABSTRACT

1b-i) Key features/functionalities/components of the intervention and comparator in the METHODS section of the ABSTRACT

We note that the intervention included: "automated smoking cessation messages tailored to individual’s stage of smoking cessation, specialized messages provided on-demand based on user requests for additional support, and a peer-to-peer social support network."

1b-ii) Level of human involvement in the METHODS section of the ABSTRACT

“Adult took part in a single individual smoking cessation counseling session.” The text message intervention is noted as being "automated," and including "a peer-to-peer social support network."

1b-iii) Open vs. closed, web-based (self-assessment) vs. face-to-face assessments in the METHODS section of the ABSTRACT

Participants were recruited from the community via offline radio and newspaper advertisements, as well as online advertisements. Outcomes were assessed via self-report questionnaires and the trial was unblinded.

1b-iv) RESULTS section in abstract must contain use data

Thirty participants were randomized each to the intervention and control groups. 83% of the intervention group and 90% of the control group completed the full 8 weeks of the intervention.

1b-v) CONCLUSIONS/DISCUSSION in abstract for negative trials

This trial was not a negative trial.

INTRODUCTION

2a-i) Problem and the type of system/solution

“It is estimated that 19.3% (45.3 million) of U.S. adults are smokers, with higher rates among younger adults and individuals with lower socioeconomic status [1]. Smoking or exposure to secondhand smoke kills more than 440,000 people in the U.S. every year, is associated with numerous chronic health conditions, and results in nearly $100 billion in healthcare costs and productivity losses annually [2,3]. Estimates suggest 69% of smokers want to quit, and in 2010 more than half of all smokers attempted to quit [1]. Although evidence-based behavioral smoking treatments exist, research suggests these resources are used by less than 10% of smokers attempting to quit [4,5]. Frequently cited barriers to utilizing behavioral interventions include the cost, time commitments, and logistics (e.g., travel, scheduling appointments) associated with these treatments [6]. To effect significant reductions in smoking rates, innovative interventions and delivery systems are needed that reach smokers effectively and efficiently [7]. “

2a-ii) Scientific background, rationale: What is known about the (type of) system

"One emerging method that may help overcome these barriers to treatment is the use of mobile communication technologies, or mHealth treatment programs. The use of mobile technology, such as cell phones, smartphones, and tablet devices to deliver health-related interventions is a rapidly expanding area of research and practice [8, 9, 10, 11]. With more than 80% of adults in the U.S. – across various demographic groups – owning mobile phones [12], the majority of smokers can be reached for smoking cessation treatment using mobile technology. Previous research suggests ("landline") telephone counseling for smoking cessation is efficacious [13], is acceptable to smokers, and in many cases is the preferred mode of treatment as compared to face-to-face behavioral counseling [13, 14].

As with landline telephones, mobile phones can be used to provide behavioral counseling, but offer the additional benefit of allowing for text messaging as well. Text message-based mHealth interventions are growing in popularly because of the ease of use and low cost of text messaging. This intervention modality also offers several benefits over face-to-face or telephone-based behavioral interventions, primarily because mobile technology allows interventions to be delivered to people in everyday settings and in real-time. This allows for the content and timing of messages to be individually tailored to individuals. For example, text messages encouraging continued abstinence from smoking can be provided at times when patients report they are in need of additional support (e.g., when having cravings, at times when they typically smoke). The use of mobile technology can allow for multiple daily contacts over longer periods of time (i.e., several text messages per day). This increased intensity and tailoring of interventions may improve adherence to self-help materials, resulting in higher quit rates [15, 16, 17, 18].

To date, there have been several interventions designed using text messaging to deliver smoking cessation interventions. Although many studies have evaluate short-term treatment outcome for smokers either using single group [19, 20] or randomized designs [21, 22, 23], there are fewer text message-based smoking cessation programs that have carefully examined long-term outcome data (>6 months) in randomized trials [24, 25]. In the past several years, two Cochrane reviews have been conducted evaluating the short-term [26] and longer-term [27] efficacy of text message-based smoking cessation interventions. The authors have concluded that although there is heterogeneity among study results, overall there is a benefit of mobile phone-based smoking cessation interventions."

METHODS

3a) CONSORT: Description of trial design (such as parallel, factorial) including allocation ratio

The goal of the present work was to develop and evaluate the feasibility and preliminary efficacy of a smoking cessation intervention program delivered through text messaging. The content and structure of this program was designed based on evidence-based intervention guidelines for smoking treatment programs, while also incorporating feedback from potential end users regarding their preferences. Formative work was conducted to assess user preferences for the intervention features, content and delivery schedules, and is reported elsewhere [28]. In the present study, we report on the intervention feasibility and acceptability of this intervention method, and present data from an initial study that included follow up assessments thorough six months post-treatment.

3b) CONSORT: Important changes to methods after trial commencement (such as eligibility criteria), with reasons

"Eligible individuals were then scheduled for an in-person orientation visit during which they were told more about the program, provided written informed consent, and took part in a single in-person smoking cessation counseling session. Following initial counseling participants were randomized to either the TXT intervention or control condition. Over a period of 3 months a total of 7 participants were enrolled and randomized using these procedures. Slow recruitment and high attrition rates prior to attending orientation prompted a change in recruitment methods.

We developed a web portal that provided the pre-screening introduction to the study. Interested individuals clicked through to a second page that presented an online screener to determine the individual's eligibility. Eligible individuals were then presented with an online consent form to sign electronically. The online consent included a brief quiz to ensure that individuals understood the primary points of the trial. After signing consent, the participant provided identifying and contact information, and completed an online baseline assessment. At the conclusion of the assessment the web program randomly assigned individuals to the study arms and presented an online Google calendar to schedule their counseling session. Fifty-one participants were recruited and randomized over 21 days using these website-based procedures."

3b-i) Bug fixes, Downtimes, Content Changes

Slow recruitment and high attrition rates prior to attending orientation prompted a change in recruitment methods.
We developed a web portal that provided the pre-screening introduction to the study. Interested individuals clicked through to a second page that presented an online screener to determine the individual's eligibility. Eligible individuals were then presented with an online consent form to sign electronically. The online consent included a brief quiz to ensure that individuals understood the primary points of the trial. After signing consent, the participant provided identifying and contact information, and completed an online baseline assessment. At the conclusion of the assessment the web program randomly assigned individuals to the study arms and presented an online Google calendar to schedule their counseling session.

4a) CONSORT: Eligibility criteria for participants

To be eligible for this study, individuals had to meet the following inclusion criteria: (1) current daily smoker, (2) interested in quitting smoking in the next 30 days, (3) have a mobile phone with text messaging capability, and (4) use text messaging at least once monthly.

4a-i) Computer / Internet literacy

To be eligible individuals had to already own a mobile phone with text messaging capability, and use text messaging at least once monthly

4a-ii) Open vs. closed, web-based vs. face-to-face assessments:

"Participants were recruited through advertisements in local media outlets (internet sites, radio programs). Interested individuals called or texted our study phone number for more information. The study Research Assistant (RA) reached callers by voice phone, provided a brief description of the study (pre-screening introduction), and screened potential participants for eligibility (see Participants section above for eligibility criteria). Eligible individuals were then scheduled for an in-person orientation visit during which they were told more about the program, provided written informed consent, and took part in a single in-person smoking cessation counseling session."
"Once enrolled, participants completed the individual counseling session and began receiving either the intervention or control text messages on their personal cell phones for the following 8 weeks. At the mid-point (week 4) and end (week 8) of the intervention all participants completed the outcome measures described above using the website. They again completed the outcome measures 3 and 6 months after completing the intervention."

4a-iii) Information giving during recruitment

The studies initial protocol utilized in person consent, before switching over to an online consent form.
"The study Research Assistant (RA) reached callers by voice phone, provided a brief description of the study (pre-screening introduction), and screened potential participants for eligibility (see Participants section above for eligibility criteria). Eligible individuals were then scheduled for an in-person orientation visit during which they were told more about the program, provided written informed consent, and took part in a single in-person smoking cessation counseling session".
"We developed a web portal that provided the pre-screening introduction to the study. Interested individuals clicked through to a second page that presented an online screener to determine the individual's eligibility. Eligible individuals were then presented with an online consent form to sign electronically. The online consent included a brief quiz to ensure that individuals understood the primary points of the trial."

4b) CONSORT: Settings and locations where the data were collected

"This study was conducted in the research facilities of the Miriam Hospital, which is affiliated with the Alpert School of Medicine at Brown University."

Additional information not in the manuscript: All electronic data are stored in password protected, secured computer systems. All paper data will be stored in a locked file cabinet. Data will only be removed when coded, entered, or audited.
The data entry system will require a login identification and password in order to gain access to the data. Where appropriate, validation and range rules will be applied to the actual entry fields. Only the Principal Investigator and co-Investigators will be able to view the data in its raw state.
All direct human subjects involvement for this study will be conducted at the Miriam Hospital (Providence, Rhode Island) performance site. Analysis of data will be performed at the Providence, Rhode Island site.

4b-i) Report if outcomes were (self-)assessed through online questionnaires

Yes, outcome assessments were conducted by online or phone interview – in both cases these are considered self-report data. "At the mid-point (week 4) and end (week 8) of the intervention all participants completed the outcome measures described above using the website. The study RA sent email reminders to participants when assessments were due reminding them to complete the online questionnaires and provided a link to the online questionnaires."

4b-ii) Report how institutional affiliations are displayed

All posted internet advertisements contained the name of the Miriam Hospital and Brown Medical School as well as their official logos. All radio ads also identified the study as being conducted at The Miriam Hospital and Brown Medical School and sponsored by the National Institutes of Health. Before being posted, all internet and radio advertisements were reviewed by the Miriam Hospital Institutional Review Board (IRB). The secured website included 3 different logos: 1) Live Inspired [the company developing the webpage], 2) the National Institutes of Health and 3) The Miriam Hospital / Brown Medical School.

5) CONSORT: Describe the interventions for each group with sufficient details to allow replication, including how and when they were actually administered

5-i) Mention names, credential, affiliations of the developers, sponsors, and owners

"Beth C. Bock,1 Kristin E. Heron,2 Ernestine G. Jennings,1 Kathleen M. Morrow,1 Victoria Cobb,3 Joshua C. Magee, 4 & Joseph L. Fava3

1 Alpert Medical School, Brown University, Centers for Behavioral and Preventive Medicine, Providence, RI, United States
2 Survey Research Center, Pennsylvania State University, University Park, PA, United States
3 The Miriam Hospital, Centers for Behavioral and Preventive Medicine, Providence, RI, United States
4 University of Cincinnati, Dept. of Family and Community Medicine, University of Cincinnati, Cincinnati, OH, United States"

"The authors have no conflicts of interest to declare in relation to this paper or the study".

5-ii) Describe the history/development process

"Formative work which included several focus groups with young adult smokers was conducted to inform the development of the intervention design and content [28]. The intervention, including both the text messages and individual counseling session, was modeled after national treatment guidelines [34], and guided by Social Cognitive Theory [35, 36] and the stages of change model [37]. All intervention content (including the text message and counseling session content) was developed and reviewed by Ph.D.-level clinical psychologists with expertise in smoking cessation treatment".

5-iii) Revisions and updating

After the initial 17 weeks into the pilot study, the recruitment process was switched to entirely web based versus traditional.

"Use of our traditional method of recruitment including in-person visits for orientation and consent together with an initial face-to-face smoking cessation counseling visit resulted in very slow recruitment and high rates of loss of eligible individuals. Both recruitment yield and speed were greatly improved when all processes were revised to be congruent with the way that the target audience uses technology".

5-iv) Quality assurance methods

"Study staff ran a series of 10 mock participants with differing answers to questions in order to test the accuracy of data collected on the web portal before launching."

At the time of collection, there was initial clerical review of all data for accuracy and completeness. Every effort will be made to ensure that missing data are kept to a minimum. Data entry programs with range checking and response validation will be used for all data keypunched.

5-v) Ensure replicability by publishing the source code, and/or providing screenshots/screen-capture video, and/or providing flowcharts of the algorithms used

We do not currently include source code or screenshots from the intervention.

5-vi) Digital preservation

Not applicable because it was not a URL-based intervention.

5-vii) Access

"Interested individuals called or texted our study phone number for more information."
"New advertisements were developed to include the option of accessing the study website directly, in addition to calling or texting our staff. "
Not in the manuscript: All participants were enrolled into the "ILiveInspired" text message service for free and received either intervention or control text messages on their personal cell phones for the following 8 weeks. Participants were also given free access to the webpage which they entered by using their personal email addresses and telephone number which was collected following the consent process.

5-viii) Mode of delivery, features/functionalities/components of the intervention and comparator, and the theoretical framework
“The intervention was designed to accommodate participants who were at various stages of quitting and to allow for different trajectories to abstinence. Four tracks were created that included “Not Ready,” “Prepare,” “Quit,” and “Relapse.” The “Not Ready” track was designed for individuals who wished to quit smoking in the next 30 days, but who were not ready to set a quit date. Messages in the “Not Ready” track consisted of once-daily messages delivered for up to 14 days and were aimed at enhancing motivation to quit. The “Prepare” track was designed for individuals who set a targeted quit day within the next 14 days, and consisted of twice-daily messages that included tips and advice on obtaining support for quitting, medications to aid quitting, dealing with nicotine addiction, coping with stress, problem solving and self-monitoring, and motivational messages. More than 200 messages were generated to address the following categories: social support, problem solving, decision making, motivational support, behavioral tips, information about smoking cessation medications, and addiction education. Participants received messages that spanned these categories to provide broad coverage of topics, and to ensure messages were not duplicated”.

“To avoid redundancy, variations on these messages were created for a “Prepare-2” track which was delivered to those who failed to quit on quit day or relapsed. The “Quit” track contained messages delivered 4 times daily for 2 weeks, then twice daily for 4 weeks (6 weeks total). These messages addressed the same general topics noted above, but were tailored to be appropriate for individuals who were currently engaged in quitting smoking”.

“At study enrollment participants randomized to the intervention (TXT) were assigned to the either the “Prepare” or “Not Ready” tracks depending on whether they set a target quit date. Those who remained in the “Not Ready” track for all 14 days without setting a quit date were called by the study counselor and encouraged to set a quit date. At an individual’s designated quit day, participants were moved into the “Quit” track. During the first week following quit day, participants answered texted questions regarding whether they had been able to quit. Those who did not quit on their quit date or who relapsed were asked whether they wished to set a new date. Those setting a new quit date within 14 days were moved into the “Prepare” track, the remaining participants were moved into the “Not Ready” track. Participants could text the key words “Prepare,” “Quit,” “Not Ready,” and “Relapse” at any time during the program to move themselves into the appropriate track for their experience with quitting. For example, an individual in “Prepare” who decided to quit several days before his designated quit day could text “Quit” to move himself immediately into that part of the program. A participant in the “Quit” part of the program could text “Relapse” if she was smoking again and could then choose to begin the “Not Ready” or “Prepare” track?”.
Additional features
“In our formative work, focus group participants strongly supported being able to receive messages “on demand” at times when they were experiencing a craving. This feature was included in the intervention; participants could text “Crave” and would receive a text message back with a tip for coping with cravings. In addition, after quitting, participants reporting that they had “slipped” and smoked a cigarette, would receive text messages immediately and twice daily for 3 days targeting coping and getting back on track with quitting. After a “slip” they were texted to report on their abstinence status and received tailored messages depending on their response”.
Comparator Condition
“The text message intervention was compared to a control condition. Participants randomly assigned to the control group (Mojo) received the same initial counseling session followed by 8 weeks of daily non-smoking-related motivational texts (e.g., “It takes just one positive step to begin the journey out of a difficult rut. Step out today!”). ”
5-ix) Describe use parameters

"Once enrolled, participants completed the individual counseling session and began receiving either the intervention or control text messages on their personal cell phones for the following 8 weeks. At the mid-point (week 4) and end (week 8) of the intervention all participants completed the outcome measures described above using the website. They again completed the outcome measures 3 and 6 months after completing the intervention."

"Individuals using the program will receive multiple daily messages at the onset of the program and gradually receive fewer as they progress through. The message program begins one week prior to the participant-determined Targeted Quit Day (TQD). During this period participants receive two messages per day designed to prepare them for making a quit attempt. On their TQD and throughout the first two weeks of being quit, participants will receive 3- 4 messages per day that provide support and encouragement and are designed to influence likely mediators of successful cessation (e.g., self-efficacy). Those who wish to receive additional messages can elect to utilize the "crave" function that triggers additional messages focusing on strategies to deal with cravings and temptations to smoke, and motivational support. Participants, who have smoked after their targeted quit day, can also use the "slip" function (by texting "slip"), which triggers additional messages focused on getting back on track before a full relapse to regular smoking occurs. At any time after quit day, participants can also text "relapse" to indicate they are smoking again and the program will re-start."

5-x) Clarify the level of human involvement

"At study enrollment, all participants also took part in an individual 30-minute counseling session led by a PhD-level clinical psychologist. Participants chose the format of the session (i.e., in person, telephone, Google Chat, or Skype), and were provided a copy of a quit smoking guide published by the American Lung Association [38]. During the session, the counselor led a discussion based upon key sections of the guide, including reasons for quitting, perceived importance of quitting and confidence in one's ability to quit, identifying obstacles to quitting, preparing for quit day, and planning strategies to aid quitting. Participants were encouraged to continue using the guide during their participation in the intervention."

Additionally: The study RA contacted participants by phone if they reported in their follow up assessments that they had been quit for 7 days.

5-xi) Report any prompts/reminders used

"The study RA sent email reminders to participants when assessments were due reminding them to complete the online questionnaires and provided a link to the questionnaires."

5-xii) Describe any co-interventions (incl. training/support)

"At study enrollment, all participants also took part in an individual 30-minute counseling session led by a PhD-level clinical psychologist. Participants chose the format of the session (i.e., in person, telephone, Google Chat, or Skype), and were provided a copy of a quit smoking guide published by the American Lung Association [38]."

6a) CONSORT: Completely defined pre-specified primary and secondary outcome measures, including how and when they were assessed

"Outcome measures were administered mid-intervention (week 4), post-intervention (week 8), and at 3-month and 6-month follow-up assessments. During these times, participants reported their smoking status, readiness and confidence in quitting (or remaining quit), and symptoms related to nicotine withdrawal. Smoking status included seven-day point prevalence abstinence, the primary outcome variable, and 24-hour point prevalence abstinence. Readiness and confidence in quitting and nicotine withdrawal symptoms were assessed using the measures described above. Feasibility and acceptability of the program were assessed by recruitment numbers, participant retention through the treatment program and final follow up assessment, and results of a program satisfaction survey."

6a-i) Online questionnaires: describe if they were validated for online use and apply CHERRIES items to describe how the questionnaires were designed/deployed

Online questionnaires were not separately validated for online use.

6a-ii) Describe whether and how "use" (including intensity of use/dosage) was defined/measured/monitored

" We found that counseling visits were accomplished by voice-phone (56%), in person (18%), Google Chat (20%), or Skype (6%). Over half (61%) of all participants used the online Google calendar to schedule their counseling session." "However, 60% of participants did not use the group messaging ("help") feature. Of those who did, 72% said it was easy or very easy to use. "

6a-iii) Describe whether, how, and when qualitative feedback from participants was obtained

Participants' feedback indicated strong appreciation of the social networking feature. As an illustrative example, one participant reported: "Yes, I think that it helps me a lot…people to talk with when you would have normally smoked a cig." Nearly one third of participants (n=17) used the peer-to-peer network for social support during their quit attempt. The number of messages sent by any one participant ranged from 2 to 177 (M = 39.7, SD = 54.0).

6b) CONSORT: Any changes to trial outcomes after the trial commenced, with reasons

No, there were no changes to the trial outcomes after the study commenced.

7a) CONSORT: How sample size was determined

7a-i) Describe whether and how expected attrition was taken into account when calculating the sample size

"The primary smoking outcome analysis examined the 7-day point prevalence abstinence from smoking using the generalized estimating equations (GEE) approach of Zeger and Liang [39] with robust standard errors, using Proc GENMOD within SAS 9.3 for Windows. The model for this outcome analysis was a 2 (treatment groups) × 3 (time points) repeated measures design that was fit using an autoregressive working correlation structure on data analyzed from an intention-to-treat (ITT) perspective in which missing participants were counted as smoking. Smoking outcomes were analyzed using odds ratios comparing TXT and Mojo (control) groups."

7b) CONSORT: When applicable, explanation of any interim analyses and stopping guidelines

This is not applicable because neither interim analyses nor stopping guidelines were used.

8a) CONSORT: Method used to generate the random allocation sequence

"Simple randomization was used to assign participants to each group via a computerized random number generator. Random assignments were placed in a sealed envelope by the study RA prior to each counseling appointment. The RA delivered the randomization assignment to the study participant immediately after completion of the counseling session."

"At the conclusion of the assessment the program used simple randomization to assign individuals to the study arms and then presented an online Google calendar to schedule their counseling session."

8b) CONSORT: Type of randomisation; details of any restriction (such as blocking and block size)

Yes, see above. No restrictions or blocking were used.

9) CONSORT: Mechanism used to implement the random allocation sequence (such as sequentially numbered containers), describing any steps taken to conceal the sequence until interventions were assigned

"Random assignments were generated by the study statistician and placed in a sealed envelop prior to each counseling appointment. The RA delivered the randomization assignment to the study participant immediately after their initial counseling session."

10) CONSORT: Who generated the random allocation sequence, who enrolled participants, and who assigned participants to interventions

"Random assignments were generated by the study statistician and placed in a sealed envelop prior to each counseling appointment. The RA delivered the randomization assignment to the study participant immediately after their initial counseling session."

11a) CONSORT: Blinding - If done, who was blinded after assignment to interventions (for example, participants, care providers, those assessing outcomes) and how

11a-i) Specify who was blinded, and who wasn't

"The study RA conducting assessments and the counselors were blind to participant randomization assignment. "

11a-ii) Discuss e.g., whether participants knew which intervention was the "intervention of interest" and which one was the "comparator"

The consent document describes the study as follows:

You are being asked to take part in a research project because you are over age 18, you use text messaging on your cell phone daily, and you want to quit smoking. The purpose of this study is to evaluate a treatment program for young-adult smokers that delivered help and advice through mobile phone text messaging. This study will enroll 50 adult smokers. This study is sponsored by the National Institute on Drug Abuse (NIDA) which is part of the National Institutes of Health (NIH).

If you take part in this study, you will be assigned randomly (like a coin toss) to a group that gets either inspirational text messages or text messages related to quitting smoking.

In addition, all participants will receive counseling for quitting smoking. Regardless of your group assignment, you will attend a single counseling session, which can be done in-person, by phone or internet (like Skype or GoogleChat). At that session you will be given a self-help booklet with information about quitting smoking and instructions about how to use the text message service to help you quit.

11b) CONSORT: If relevant, description of the similarity of interventions

Yes: “At study enrollment, all participants also took part in an individual 30-minute counseling session led by a PhD-level clinical psychologist. Participants chose the format of the session (i.e., in person, telephone, Google Chat, or Skype), and were provided a copy of a quit smoking guide published by the American Lung Association [38].”

12a) CONSORT: Statistical methods used to compare groups for primary and secondary outcomes

“Statistical Analyses

Frequency distributions, means and standard deviations were used to characterize the overall sample. Chi-square tests and analysis of variance was used to compare groups for comparability on baseline demographic and smoking history variables. The primary smoking outcome analysis examined the 7-day point prevalence abstinence from smoking using the generalized estimating equations (GEE) approach of Zeger and Liang [39] with robust standard errors, using Proc GENMOD within SAS 9.3 for Windows. The model for this outcome analysis was a 2 (treatment groups) × 3 (time points) repeated measures design that was fit using an autoregressive working correlation structure on data analyzed from an intention-to-treat (ITT) perspective in which missing participants were counted as smoking. Smoking outcomes were analyzed using odds ratios comparing TXT and Mojo (control) groups. A secondary smoking outcome analysis also used the GEE methodology to examine 24-hour point prevalence abstinence. Further secondary analyses used analysis of variance to evaluate for change in several potential mediating variables at 8-week, 3-month, and 6-month follow-up, among respondents who provided complete data at those assessment points.”

12a-i) Imputation techniques to deal with attrition / missing values

GEE procedures and the use of intention-to-treat analyses make the imputation of missing data unnecessary.

12b) CONSORT: Methods for additional analyses, such as subgroup analyses and adjusted analyses

No sub-group or adjusted analyses were conducted.

RESULTS

13a) CONSORT: For each group, the numbers of participants who were randomly assigned, received intended treatment, and were analysed for the primary outcome

“Over a period of 3 months a total of 7 participants were enrolled and randomized using these procedures. Slow recruitment and high attrition rates prior to attending orientation prompted a change in recruitment methods.”

“After implementation, 51 participants were recruited and randomized over 21 days using these website-based procedures. “

“The model for this outcome analysis was a 2 (treatment groups) × 3 (time points) repeated measures design that was fit using an autoregressive working correlation structure on data analyzed from an intention-to-treat (ITT) perspective in which missing participants were counted as smoking.”

“Table 1: Smoking Status by Group for 7-Day and 24-Hour ITT at 8 Weeks, 3 Months and 6 Months

Total Txt2Quit Mojo
Sample Size603030

“

13b) CONSORT: For each group, losses and exclusions after randomisation, together with reasons

Diagram illustrates the losses and exclusions after randomization.

13b-i) Attrition diagram

Not applicable. The study did not involve use of a website except for assessment surveys. The number of logins for assessments is reflected in the number of completed follow up assessments. There is no method for assessing whether participants read text messages sent to them, however all messages were sent.

14a) CONSORT: Dates defining the periods of recruitment and follow-up

Participants were recruited between January and June 2011.

14a-i) Indicate if critical “secular events” fell into the study period

No secular events took place during the trial.

14b) CONSORT: Why the trial ended or was stopped (early)

No. The trial was not ended or stopped early.

15) CONSORT: A table showing baseline demographic and clinical characteristics for each group

Baseline demographics and clinical characteristics are presented in text format.

15-i) Report demographics associated with digital divide issues

"Participants were primarily non-Hispanic white (66%) or Black (19%), with 7% bi/multi-racial or unsure, 2% Hispanic White, and 7% who did not respond. With respect to ethnicity 19% were Hispanic/Latino. Most had completed some college (36%) or had graduated from college (26%), with 21% graduating from high school, while 9% had not graduated from high school, and 9% did not respond. Most participants (35%) worked part time (<35 hrs/week) or full time (31%), with 26% unemployed and 9% who did not respond. Total household income was less than $25,000 for half (50%) of the participants. On average, participants were 16.0 years old (SD=2.9) when they first started regular (daily) cigarette smoking. At the start of the study, participants smoked an average of 16.3 cigarettes/day (SD=8.3; range 4-40). Participants had made an average of 4.1 (SD= 3.8) serious quit attempts in their lives. Of the 53 participants who responded to a temporal intention to quit item, 94.3% noted at baseline that they were planning to quit in the next 30 days. Baseline FTND scores averaged 4.9 (SD=2.5), suggesting moderate nicotine dependence. Baseline CESD scores averaged 10.4 (SD=5.9), and over one third of participants (35%) had CESD scores above 11, indicative of significant levels of depressive symptoms [33]."

16a) CONSORT: For each group, number of participants (denominator) included in each analysis and whether the analysis was by original assigned groups

16-i) Report multiple "denominators" and provide definitions

The analysis used GEE methods in which missing participants are counted as smokers (intention-to-treat). Thus, all randomized participants are included in every analysis. This information is presented in the "Statistical Analyses" section of the paper.

16-ii) Primary analysis should be intent-to-treat

Yes, see above.

17a) CONSORT: For each primary and secondary outcome, results for each group, and the estimated effect size and its precision (such as 95% confidence interval)

"A 2 (treatment groups) × 3 (time points) GEE repeated measures analysis examined the effects of the TXT intervention vs. Mojo for differences in 7-day point prevalence abstinence. There was a significant main effect for treatment group (p = .02) with higher odds of 7-day point prevalence abstinence for the TXT group compared to the Mojo group (OR = 4.52, 95% CI = 1.24, 16.53). There were no significant effects for time (p = .34) or the time × treatment group interaction (p = .60). While the overall main effect was significant, contrast estimates did not find specific differences between TXT vs. Mojo at week 8 (23.3% vs. 10.7%, OR = 2.54, 95% CI = 0.59, 10.99), month 3 (16.7% vs. 3.6%, OR = 5.40, 95% CI = 0.59, 49.47), or month 6 (20.0% vs. 3.6%, OR = 6.75, 95% CI = 0.76, 60.15), likely due to reduced statistical power at any individual time point. We also evaluated for treatment group differences in 24-hour point prevalence in a similar manner, using a 2 (treatment groups) × 3 (time points) GEE repeated measures analysis, but found no significant effects for group (p = .11), time (p = .23), or the time × treatment group interaction (p = .88)".

17a-i) Presentation of process outcomes such as metrics of use and intensity of use

"End of treatment (week 8) satisfaction ratings showed that nearly all participants said they were satisfied (41%) or very satisfied (48%) with the program, and 63% said the program met their expectations. Most participants said the program was helpful (40%) or very helpful (45%). However, 60% of participants did not use the group messaging ("help") feature. Of those who did, 72% said it was easy or very easy to use. Participants' feedback indicated strong appreciation of the social networking feature. As an illustrative example, one participant reported: "Yes, I think that it helps me a lot…people to talk with when you would have normally smoked a cig." Nearly one third of participants (n=17) used the peer-to-peer network for social support during their quit attempt. The number of messages sent by any one participant ranged from 2 to 177 (M = 39.7, SD = 54.0). "

17b) CONSORT: For binary outcomes, presentation of both absolute and relative effect sizes is recommended

Yes. The number of individuals quit and percent are given in the results in text and in Table 1.

18) CONSORT: Results of any other analyses performed, including subgroup analyses and adjusted analyses, distinguishing pre-specified from exploratory

Sub-group analyses and adjusted analyses were not performed.

18-i) Subgroup analysis of comparing only users

Not done – intention to treat analyses are the most conservative and count all randomized participants.

19) CONSORT: All important harms or unintended effects in each group

No harms or unintended effects were observed.

19-i) Include privacy breaches, technical problems

No privacy breaches or technical problems occurred.

19-ii) Include qualitative feedback from participants or observations from staff/researchers

Limited qualitative feedback was obtained from participants using consumer satisfaction measures and is included in the text. "End of treatment (week 8) satisfaction ratings showed that nearly all participants said they were satisfied (41%) or very satisfied (48%) with the program, and 63% said the program met their expectations. Most participants said the program was helpful (40%) or very helpful (45%). However, 60% of participants did not use the group messaging ("help") feature. Of those who did, 72% said it was easy or very easy to use. Participants' feedback indicated strong appreciation of the social networking feature. As an illustrative example, one participant reported: "Yes, I think that it helps me a lot…people to talk with when you would have normally smoked a cig." Nearly one third of participants (n=17) used the peer-to-peer network for social support during their quit attempt. The number of messages sent by any one participant ranged from 2 to 177 (M = 39.7, SD = 54.0). "

DISCUSSION

20) CONSORT: Trial limitations, addressing sources of potential bias, imprecision, multiplicity of analyses

20-i) Typical limitations in ehealth trials

"At the same time, there were also limitations that can be addressed by future research. First, results from pilot studies are inherently less reliable due to the small number of participants [41], therefore these findings will need replication in a larger trial to verify reliability of the results. Second, given that this was a pilot study, we did not biochemically verify abstinence from smoking, but instead relied on self-report. When this and other interventions are tested in larger clinical trials, objective measures of outcome should be used in order to reduce biases associated with self-reported abstinence from smoking. Third, larger scale studies are needed to provide sufficient statistical power to examine factors that may act as mechanisms of action as well as factors that may moderate intervention efficacy. Fourth, more thorough integration of peer-to-peer social networking within a text message intervention could further enhance engagement with and benefits resulting from this type of treatment."

21) CONSORT: Generalisability (external validity, applicability) of the trial findings

21-i) Generalizability to other populations

As this study was not setting specific, and was a pilot study, we did not discuss issues of generalizability.

21-ii) Discuss if there were elements in the RCT that would be different in a routine application setting

Routine administration of this intervention would not involve major changes. The only difference would be the elimination of study assessments.

22) CONSORT: Interpretation consistent with results, balancing benefits and harms, and considering other relevant evidence

22-i) Restate study questions and summarize the answers suggested by the data, starting with primary outcomes and process outcomes (use)

"Although this study was designed to develop and provide initial testing of the TXT-2-Quit system, significant differences were found between treatment groups, with individuals randomized to the intervention group showing higher point-prevalence abstinence rates versus the comparison group. These results are especially encouraging given that all control participants received an individual smoking cessation counseling session, and at least one daily text message for the duration of the 8-week intervention."

22-ii) Highlight unanswered new questions, suggest future research

"Future work that is statistically powered for secondary outcome variables should more vigorously evaluate factors that could serve as mediators or moderators of treatment efficacy, including self-efficacy for quitting, nicotine withdrawal symptoms, readiness for change, and depression."

"A peer-to-peer network, which allowed participants to communicate and encourage each other, was created at the behest of focus group participants. However, we found that this feature was used by less than half of study participants. As designed, the social support feature was only accessed by individuals who requested "help" from others. It may be that some individuals do not need additional social support through this type of intervention or that they already receive (or think they receive) sufficient support from other social networks. Some people may also be hesitant to admit they need help, or tend to underestimate the degree to which seeking help from peers would improve their chances of quitting. This may need to be tested by making the social support network a more integrated (non-optional) part of the intervention in order to better evaluate the utility of including peer networks in text message-based smoking cessation programs. "

Other information

23) CONSORT: Registration number and name of trial registry

Yes. The trial registration number is posted in the abstract

24) CONSORT: Where the full trial protocol can be accessed, if available

The trial protocol is described in this paper. Items such as the website programming code, is proprietary.

25) CONSORT: Sources of funding and other support (such as supply of drugs), role of funders

Sources of funding are identified in the manuscript. "Funding for this study was provided by a grant (R21 DA027142) from the National Institute on Drug Abuse (NIDA) to Dr. Bock"

X26-i) Comment on ethics committee approval

"This study conducted in the research facilities of the Miriam Hospital, which is affiliated with the Alpert School of Medicine at Brown University and was approved by the Institutional Review Board prior to initiating recruitment."

x26-ii) Outline informed consent procedures

Consent was conducted both off and online.

"The study Research Assistant (RA) reached callers by voice phone, provided a brief description of the study (pre-screening introduction), and screened potential participants for eligibility (see Participants section above for eligibility criteria). Eligible individuals were then scheduled for an in-person orientation visit during which they were told more about the program, provided written informed consent, and took part in a single in-person smoking cessation counseling session. "

"We developed a web portal that provided the pre-screening introduction to the study. Interested individuals clicked through to a second page that presented an online screener to determine the individual's eligibility. Eligible individuals were then presented with an online consent form to sign electronically. The online consent included a brief quiz to ensure that individuals understood the primary points of the trial."

X26-iii) Safety and security procedures

As this was a minimal-risk study, no additional safety procedures (e.g., hotline) were implemented. Data were collected by phone interview and/or by online secure web-portal. All study data were coded with a participant ID number and the link between identifying information and the ID number was stored in a secure, password-protected database with limited access entry by authorize study staff. These details were not included in the manuscript to avoid excessive length.

X27-i) State the relation of the study team towards the system being evaluated

The authors have no conflicts of interest to declare in relation to this paper or the study."
